# Assessment of Antiviral Coatings for High-Touch Surfaces by Using Human Coronaviruses HCoV-229E and SARS-CoV-2

**DOI:** 10.1128/AEM.01098-21

**Published:** 2021-09-10

**Authors:** S. Butot, L. Baert, S. Zuber

**Affiliations:** a Société des Produits Nestlé, Nestlé Research, Institute of Food Safety and Analytical Science, Lausanne, Switzerland; Centers for Disease Control and Prevention

**Keywords:** SARS-CoV-2, antiviral activity, antiviral coating, human coronavirus 229E, viral log reduction

## Abstract

A novel and robust approach to evaluate the antiviral activity of coatings was developed, assessing three commercially available leave-on surface coating products for efficacy against human coronaviruses (HCoVs) HCoV-229E and severe acute respiratory syndrome coronavirus 2 (SARS-CoV-2). The assessment is based on three criteria that reflect real-life settings, namely, (i) immediate antiviral effect, (ii) effect after repeated cleaning of the coated surface, and (iii) antiviral activity in the presence of organic material. The results showed that only a copper compound-based coating successfully met all three criteria. A quaternary ammonium compound-based coating did not meet the second criterion, and a coating based on reactive oxygen species showed no antiviral effect. Moreover, the study demonstrated that HCoV-229E is a relevant SARS-CoV-2 surrogate for such experiments. This new approach allows benchmarking of currently available antiviral coatings and future coating developments to avoid unjustified claims. The deployment of efficient antiviral coatings can offer an additional measure to mitigate the risk of transmission of respiratory viruses like SARS-CoV-2 or influenza viruses from high-touch surfaces.

**IMPORTANCE** SARS-CoV-2, the virus responsible for the coronavirus disease 2019 (COVID-19) pandemic, is transmitted mainly person-to-person through respiratory droplets, while the contribution of fomite transmission is less important than suspected at the beginning of the pandemic. Nevertheless, antiviral-coating solutions can offer an additional measure to mitigate the risk of SARS-CoV-2 transmission from high-touch surfaces. The deployment of antiviral coatings is not new, but what is currently lacking is solid scientific evidence of the efficacy of commercially available self-disinfecting surfaces under real-life conditions. Therefore, we developed a novel, robust approach to evaluate the antiviral activity of such coatings, applying strict quality criteria to three commercially available products to test their efficacies against SARS-CoV-2. We also showed that HCoV-229E is a relevant surrogate for such experiments. Our approach will also bring significant benefit to the evaluation of the effects of coatings on the survival of nonenveloped viruses, which are known to be more tolerant to desiccation and disinfectants and for which high-touch surfaces play an important role.

## INTRODUCTION

The first reported cases of severe acute respiratory syndrome coronavirus 2 (SARS-CoV-2) pneumonia occurred in Wuhan, Hubei Province, China, in December 2019 and January 2020 (1, 2), and its spread rapidly developed into the coronavirus disease 2019 (COVID-19) worldwide pandemic (3). The main transmission route of SARS-CoV-2 is human-to-human by close contact through respiratory droplets and possibly aerosols (4). The persistence of infectious SARS-CoV-2 was shown to be high, up to 4 days, on various surfaces, such as stainless steel, plastic, and glass, with infectivity better preserved in the presence of proteins (5, 6). Surfaces in hospital and community settings have been shown to be widely contaminated by SARS-CoV-2 RNA (7–9). SARS-CoV-2 and other coronaviruses, such as human coronavirus 229E (HCoV-229E), as well as influenza viruses, are efficiently and rapidly inactivated by alcohol solutions and disinfectants used for routine cleaning and sanitation (10–13), but chemical disinfectants are relatively short lived, for example, due to evaporation in the case of alcohol. As an additional measure in the cleaning regime, antiviral coatings can contribute to the hygiene of high-touch surfaces. Modification and/or functionalization of surfaces (sometimes called “self-disinfecting surfaces” or coatings) to quickly inactivate microorganisms upon contact is a highly relevant research area (14–16).

A number of commercially available coatings advertise antiviral properties; however, laboratory evidence demonstrating efficacy is mostly lacking. A robust methodology that mimics real-life conditions is urgently needed to evaluate the antiviral claims of such products.

In this study, we provide a new approach comprised of three criteria to evaluate the antiviral potential of a surface coating, namely, (i) immediate antiviral activity, (ii) antiviral activity of the coating after repeated cleaning, and (iii) the effect of organic material deposited by finger contact on the antiviral activity of the coating. We tested the approach with SARS-CoV-2 and with HCoV-229E as a potential surrogate using three available commercial products claiming antiviral effects based on distinct effector mechanisms, i.e., reactive oxygen species (ROS), copper compounds, and quaternary ammonium compounds (QACs).

## RESULTS AND DISCUSSION

A systematic approach to evaluate the antimicrobial activity of coatings is currently lacking (17). Standards like the American Society of Testing and Materials (ASTM) method E1053 (18) and ISO 21702 (19) to assess antiviral activity on nonporous surfaces only consider the immediate antiviral activity, which is insufficient; hence, our objective was to develop a testing protocol with additional and meaningful hurdles that closely reflect real-life surface exposure. We developed a comprehensive approach using three criteria to evaluate the antiviral potentials of different surface coatings. First, a protocol was established to evaluate the immediate antiviral activity, based on the experimental setup of ISO 21702 but with modifications to better represent real-life settings (19). The most important modification was to air dry the inoculum for 15 min instead of covering it with a cover film that keeps it wet. This allowed consideration of potential viral inactivation due to simple drying on the surface, as the moisture of a droplet will in most cases have evaporated when the next person touches the surface. The second modification was that the contact times were shortened from 24 h to 0, 30, or 120 min. Indeed, an antiviral coating is only efficient if it reduces the viral load quickly, as there is potentially only a very short interval between users of high-touch surfaces. In addition to the immediate antiviral activity, two other key aspects were included in the evaluation, namely, the robustness toward cleaning and the inherent capacity of the coating to work despite the presence of organic material.

### Immediate antiviral activity.

**FIG 1:**
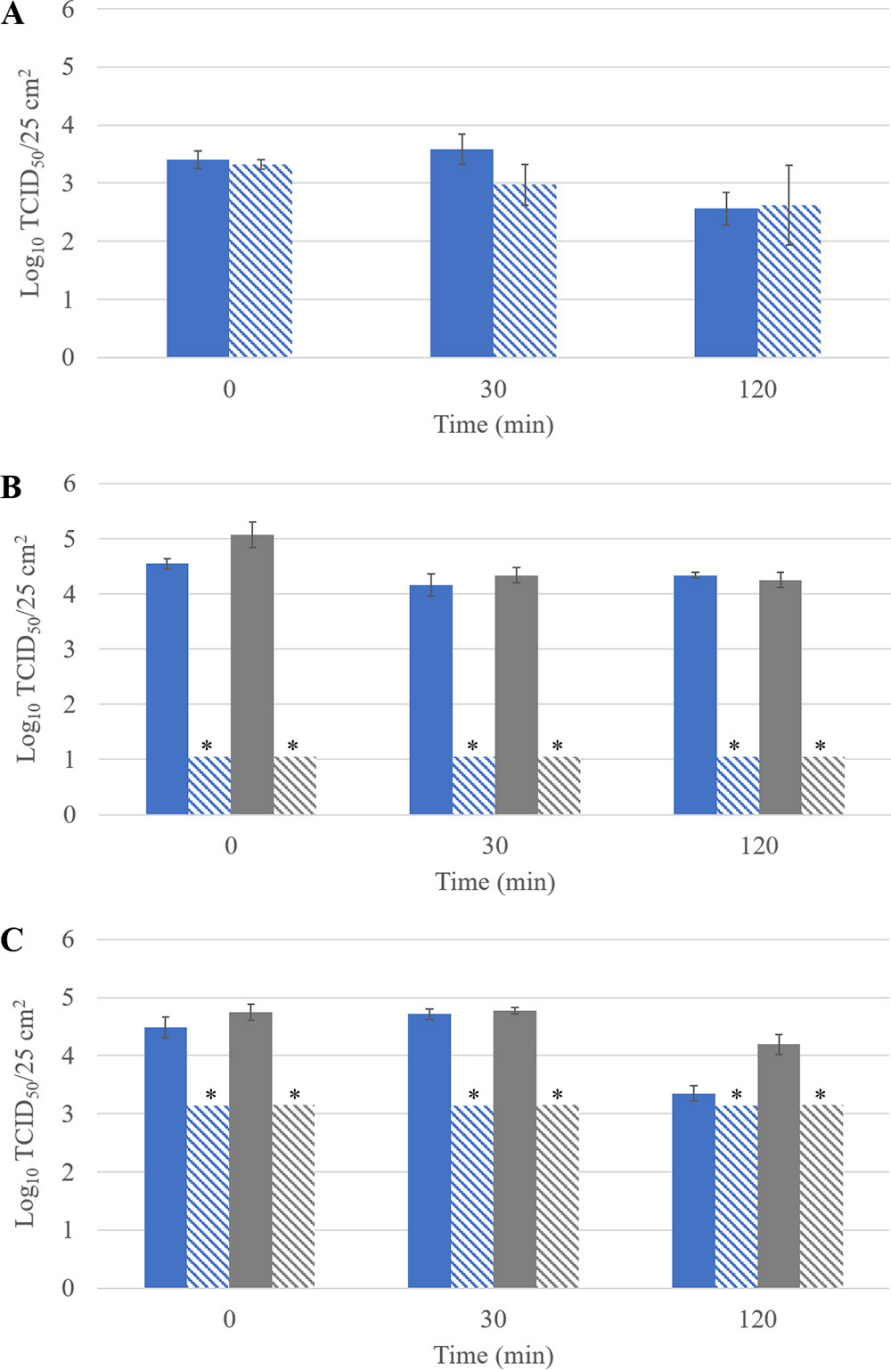
Survival of HCoV-229E (blue) and SARS-CoV-2 (gray) on noncoated surfaces (solid bars) and on coated surfaces (hatched bars) after 0, 30, or 120 min of contact time at room temperature. (A) ROS-based coating; (B) copper compound-based coating; (C) QAC-based coating. Time zero corresponds to 15 min drying after spiking of the virus on the surfaces. Asterisks highlight log_10_ values below the LOQ. The LOQ for both viruses was 1.05 log_10_ TCID_50_/25 cm^2^ for the copper compound-based coating, whereas the QAC-based coating induced cytotoxicity on the two cell lines, increasing the LOQ for both viruses to 3.15 log_10_ TCID_50_/25 cm^2^. Error bars represent the standard deviations; *n* = 3.

The immediate antiviral activity of the three coatings was evaluated by comparing the survival of HCoV-229E on noncoated versus coated surfaces for each contact time (0, 30, or 120 min) at room temperature (Fig. 1). At time zero (corresponding to 15 min of drying after spiking of the virus on the surfaces), no reduction of HCoV-229E was obtained with the coating based on ROS (Fig. 1A), whereas the coatings based on copper compounds and on QACs inactivated HCoV-229E by more than 3.5 and 2.0 log_10_, respectively (Fig. 1B and C). At times 30 and 120 min, the ROS-based coating showed low (0.6 log_10_) and no antiviral activity, respectively (Fig. 1A). The ROS-based coating, when activated by light, forms ROS with the moisture in the air. It is possible that we did not observe viral inactivation because no ROS were formed or that the ROS did not affect HCoV-229E within the time span of 2 h. Another study using TiO_2_-coated glass observed more than 3 log_10_ reduction of influenza virus, but only after 4 h of UVA exposure (20). Based on the results obtained with HCoV-229E, the ROS-based coating was not investigated any further.

The two coatings which showed immediate antiviral activity against HCoV-229E were evaluated for antiviral activity using SARS-CoV-2 after 0, 30, or 120 min of contact time at room temperature (Fig. 1B and C). At time zero, reductions of more than 4.0 log_10_ and more than 1.6 log_10_ of SARS-CoV-2 were observed on the copper compound-based and the QAC-based coating, respectively (Fig. 1B and C).

The antiviral effect of copper has previously been reported for HCoV-229E and SARS-CoV-2 (21, 22). The antiviral activity of the copper compound-based coating used in our study is thought to be caused by contact between the surfaces of the virus and the copper compound, causing denaturation of biomolecules (e.g., proteins) that results in viral inactivation. Comparable viral inactivation was described previously, where a 30-minute exposure to Cu_2_O, another copper compound, led to a reduction of more than 5 log_10_ of bacteriophage Qβ, a small, single-stranded RNA virus (23). The mechanism of the QAC-based coating technology is based on positively charged quaternized nitrogen and carbon chain “spikes.” The negatively charged microbial cell wall of bacteria is attracted to the spikes and consequently disrupted, leading to inactivation. The antiviral activity we observed may be the result of a similar mechanism, since SARS-CoV-2 virus particles are mostly negatively charged at neutral pH (24). QACs coated on glass were also shown to be effective against influenza virus (25). It is important to mention that we observed an immediate antiviral activity only if the QAC-based coating was applied by spraying without subsequent wiping. No immediate antiviral activity was observed when the coating was sprayed on the surface directly followed by wiping to evenly distribute the product on the surface (data not shown).

### Antiviral activity after repeated cleaning.

**FIG 2:**
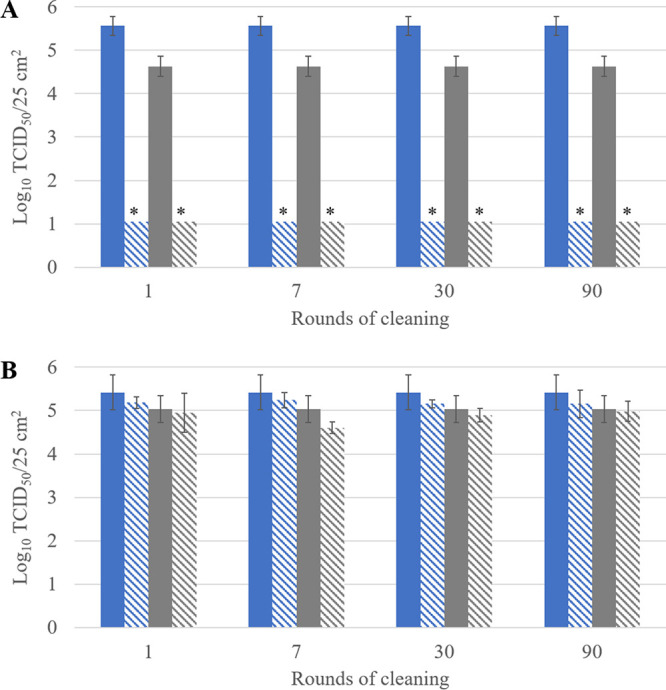
Survival of HCoV-229E (blue) and SARS-CoV-2 (gray) on noncoated surfaces (solid bars) and on coated surfaces (hatched bars) after 1, 7, 30, or 90 rounds of cleaning with a water-based detergent using a microfiber cloth. (A) Copper compound-based coating; (B) QAC-based coating. Asterisks highlight log_10_ values below the LOQ. Error bars represent the standard deviations; *n* = 3.

**FIG 3:**
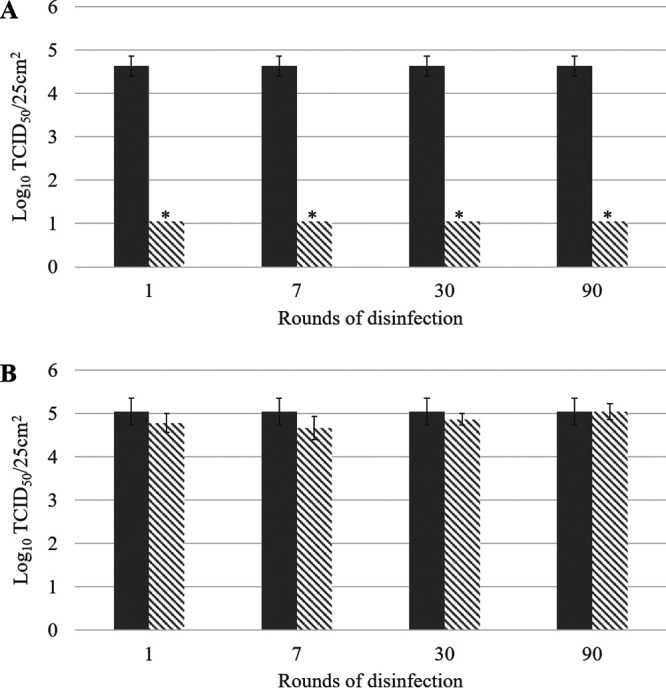
Survival of SARS-CoV-2 on noncoated surfaces (solid bars) and on coated surfaces (hatched bars) after 1, 7, 30, or 90 rounds of disinfection with 70% ethanol using a microfiber cloth. (A) Copper compound-based coating; (B) QAC-based coating. Asterisks highlight log_10_ values below the LOQ. Error bars represent the standard deviations; *n* = 3.

The antiviral activity of the coatings based on copper compounds and QACs was evaluated by cleaning the surfaces 1, 7, 30, or 90 times using a microfiber cloth with a water-based detergent. This represents an accelerated protocol to simulate 1, 7, 30, or 90 rounds of cleaning. The antiviral activity was assessed by comparing the survival of HCoV-229E and SARS-CoV-2 on noncoated surfaces versus coated and cleaned surfaces (Fig. 2). The antiviral activity of the copper compound-based coating remained intact for at least 90 rounds of cleaning (Fig. 2A), whereas the antiviral activity of the QAC-based coating was removed after only one round of cleaning (Fig. 2B). The coating sprayed on the surface was probably wiped off during cleaning. This is similar to the results from a controlled trial in a hospital setting and shows that the mode of application of a spray coating is pivotal, making it potentially less reliable than a ready-to-use adhesive film (26). Similar to the cleaning, disinfection with 70% ethanol did not affect the antiviral efficiency of the copper compound-based coating, whereas the antiviral activity was lost for the QAC-based coating (Fig. 3). These study results are necessary to define cleaning instructions (e.g., type of cloth and frequency) for the applied coating to ensure sustained antiviral activity.

### Effect of organic material introduced by finger touching.

**FIG 4:**
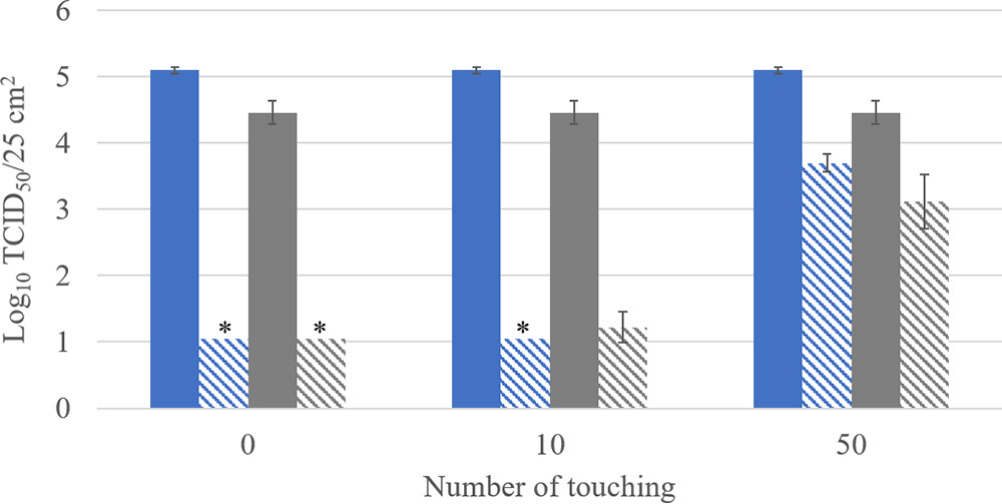
Survival of HCoV-229E (blue) and SARS-CoV-2 (gray) on noncoated surfaces (solid bars) and on surfaces with copper compound-based coating (hatched bars) that were finger touched 0, 10, or 50 times prior to virus inoculation. Asterisks highlight log_10_ values below the LOQ. Error bars represent the standard deviations; *n* = 3.

The copper compound-based coating successfully passed the two first criteria and was further evaluated for the third criterion. To assess this criterion, coated surfaces were finger touched 10 or 50 times prior to virus inoculation, to simulate the daily use of a high-touch surface (e.g., touch screens of vending machines). This experimental setup allowed the effect on antiviral activity of organic material like fingermark residues to be evaluated. The antiviral activity was assessed by comparing the survival of HCoV-229E and SARS-CoV-2 on noncoated versus coated and touched surfaces (Fig. 4). The antiviral activity of the copper compound-based coating was still high after 10 touches (>4.0 log_10_ reduction of HCoV-229E and 3.2 log_10_ reduction of SARS-CoV-2) but lower after 50 touches (1.4 log_10_ reduction of HCoV-229E and 1.3 log_10_ reduction of SARS-CoV-2). Similar log_10_ reductions were obtained for HCoV-229E and SARS-CoV-2 after 50 finger touches (*P* = 0.83). Fifty touches corresponds to the daily touching frequency of a highly used vending machine and shows that the copper compound-based coating may retain activity for roughly 1 day. Afterwards, cleaning is required to remove traces of organic material. Repeated cleaning with a microfiber cloth did not affect the antiviral activity, as shown when the second criterion was evaluated (Fig. 2A). The commercial copper compound-based coating fulfilled the three evaluation criteria and can be considered an efficient antiviral coating.

### Comparison of HCoV-229E and SARS-CoV-2.

This study assessed the antiviral activity of coatings using two viruses, HCoV-229E and SARS-CoV-2. Both viruses are human respiratory pathogens. They belong to the family *Coronaviridae* and have single-strand, positive-sense RNA genomes of approximately 26 to 32 kb in size and similar structures with spike projections from the virus membrane (27, 28). Despite these similarities, HCoV-229E cannot serve as a legitimate surrogate for SARS-CoV-2 without comparison and calibration (29). Since the limit of quantification (LOQ) was reached, the maximum log_10_ reduction was obtained for both viruses when testing the immediate antiviral activity, the first criterion, of the copper compound- and QAC-based coatings. The maximum log_10_ reduction was also observed for both viruses on the copper compound-based coating after repeated cleaning (second criterion). No log_10_ reduction was observed for either virus on the QAC-based coating after repeated cleaning. The evaluation of the effect of organic material introduced by finger touching in the third criterion also showed similar behaviors of SARS-CoV-2 and HCoV-229E. After 50 finger touches, the LOQ was not reached, allowing calculation of the *P* value (*P* = 0.83), which indicated that the log_10_ reductions obtained for the two viruses were not significantly different. Together, these results show that both viruses behaved similarly in all experiments representing the three evaluation criteria (immediate antiviral activity, antiviral activity after repeated cleaning, and the effect of organic material introduced by finger touching), demonstrating that HCoV-229E is a relevant SARS-CoV-2 surrogate for the evaluation of these surface coating products. Generating data with human coronavirus surrogates that can be handled in biosafety level 2 (BSL-2) laboratories is important, as studies with SARS-CoV-2 must be conducted in BSL-3 facilities, limiting the number of laboratories available.

In conclusion, a harmonized protocol will allow regulators and users to evaluate claims related to antiviral surfaces. It would be of interest to further elucidate the mode of action of these surfaces, especially when in contact with organic material and when exposed to extreme temperatures and pH conditions. It will also be useful to benchmark the antiviral activities of currently available coatings against those of novel technical solutions. Microbial tolerance to biocidal compounds present in coatings is unlikely due to the multitarget nature or nonspecific action of the chemicals used, as described for QACs (30). The coated surfaces will be cleaned regularly, thus avoiding long-term exposure and potential microbial tolerance to these biocidal compounds.

In the future, our approach to evaluate and verify the antiviral activity of coatings could be expanded to also encompass the effect on nonenveloped viruses, which are known to be more tolerant of desiccation and disinfectants, for example, noroviruses, which are transmitted by the fecal-oral route and for which high-touch surfaces play an important role.

## MATERIALS AND METHODS

### Virus and preparation of suspension.

HCoV-229E (ATCC VR-740) and SARS-CoV-2, kindly provided by Isabella Eckerle (Geneva University Hospitals, Center for Emerging Viral Diseases), were propagated, assayed, and titrated on human lung fibroblast MRC-5 cells (ATCC CCL-171) and on African green monkey kidney Vero C1008 (Vero 76, clone E6, Vero E6) (ECACC 85020206) cells, respectively, as described previously for enteric viruses (31). Briefly, the cells were passaged in Eagle’s minimum essential medium (EMEM) (30-2003; ATCC) supplemented with 10% fetal bovine serum (FBS) (30-2020; ATCC) and 1% penicillin/streptomycin (100×) (P0781; Sigma), followed by incubation at 37°C with 5% CO_2_. Viruses were propagated on their respective host cells, followed by incubation at 35°C with 5% CO_2_ for 1 to 2 h to allow the adsorption of the viruses to the cells. The adsorption was stopped by adding 25 ml of EMEM supplemented with 2% FBS and 1% penicillin/streptomycin (100×), followed by incubation at 35°C with 5% CO_2_. Viral stocks were purified and concentrated by polyethylene glycol precipitation (0.25 volume of 5× polyethylene glycol/NaCl solution) as described in ISO 15216 (32). The pellets were resuspended in phosphate-buffered saline (PBS) (D8662; Sigma). Viral titers, determined as the 50% tissue culture infective dose (TCID_50_) per milliliter as described previously (31), were 7.0 ± 0.3 log_10_ TCID_50_/ml (mean ± standard deviation) for SARS-CoV-2 and ranged from 6.5 ± 0.1 log_10_ TCID_50_/ml to 7.0 ± 0.1 log_10_ TCID_50_/ml for HCoV-229E.

### Antiviral-coating solutions.

ROS-based coated (the type of ROS is not described by the supplier) and noncoated 25-cm^2^ glass surfaces, copper compound-based coated and noncoated 25-cm^2^ polyethylene terephthalate (PET) films, and QAC-based spray were kindly provided by the suppliers and are commercially available as Kastus glass cover commercialized by Kastus (Dublin, Ireland), Nanoshield commercialized by Nanoveu Limited (Subiaco, Australia), and Zoono Microbe Shield (Z-71) spray commercialized by Zoono Group (Auckland, New Zealand), respectively. The ROS-based glass and copper compound-based PET film are ready-to-employ coatings to be applied like a phone screen protector. The ROS-based coating forms ROS with the moisture in the air when activated by light. The QAC-based coating needs to be sprayed on the surface of interest by the customer. In our study, this coating was sprayed on 25-cm^2^ poly(methyl methacrylate) surfaces and distributed on the whole surface using the side of a micropipette tip, followed by drying in a biosafety cabinet for at least 10 min.

### Evaluation of the antiviral activity.

The experimental setup was based on the ISO 21702 method (19) with slight modifications. The inoculum was dried for 15 min instead of covering it with a cover film that keeps it wet, and the contact times were shortened from 24 h to 0, 30, and 120 min, as the antiviral activity needs to be fast for high-touch surfaces to ensure inactivation between users.

### (i) Immediate antiviral activity.

The immediate antiviral activity against HCoV-229E and SARS-CoV-2 was evaluated by comparing the survival of the viruses on noncoated surfaces and coated surfaces after 0, 30, or 120 min of contact time at room temperature.

### (ii) Antiviral activity after repeated cleaning.

The antiviral activity against HCoV-229E and SARS-CoV-2 was evaluated by comparing the survival of the viruses on noncoated surfaces versus coated surfaces previously cleaned 1, 7, 30, or 90 times using a microfiber cloth over 5 days at room temperature. As one cleaning per day is a standard procedure for many high-touch surfaces, this protocol simulates 1 day, 1 week, 1 month and 3 months of cleaning, respectively. Cleaning was carried out with Suma Star D1 detergent (10 to 20% sodium dodecylbenzene sulfonate, 5 to 10% sodium lauryl ether sulfate, 1 to <3% ethyl alcohol) according to the supplier’s recommendations (Diversey Europe, Münchwilen, Switzerland) or with 70% ethanol for disinfection.

### (iii) Organic material effect introduced by finger touching.

The antiviral activity against HCoV-229E and SARS-CoV-2 was determined by comparing the survival of the viruses on noncoated surfaces versus coated surfaces finger touched 0, 10, or 50 times by 10 volunteers, meaning 0, 1, or 5 finger touches per person per 25 cm^2^, respectively. This corresponds to a medium (10) and high (50) daily touching frequency of a high-touch surface. The volunteers were asked to not wash or disinfect their hands prior to the finger touching. Each finger touching was performed using 3 fingers applied several times on the surface in order to cover the 25 cm^2^.

### Virus inoculation on noncoated and coated surfaces.

One hundred microliters of HCoV-229E (5.5 or 6.0 log_10_ TCID_50_) or SARS-CoV-2 (6.0 log_10_ TCID_50_), which corresponds to viral loads in saliva of infected patients (33, 34), was spread on a 25-cm^2^ noncoated or coated surface and dried for 15 min in a biosafety cabinet at room temperature. According to visual inspection, 15 min was the minimum time required to have a dry inoculum on the different surfaces employed in this study.

### Virus recovery from noncoated and coated surfaces.

Viruses were recovered by intensively swabbing the surface using a cotton-tipped swab (115-1881; VWR) predipped in Dey-Engley neutralizing broth (D3435; Sigma) diluted 5-fold in PBS (D8537; Sigma). The swab was transferred to a 1.5-ml tube containing 0.5 ml of Dey-Engley neutralizing broth diluted 5-fold in PBS. The plastic part of the swab was cut in order to close the tube, and the tube was vortexed vigorously for 1 min to release the viruses. The recovered viruses were serially diluted 5-fold and enumerated by determining the TCID_50_ (31). Preliminary experiments demonstrated that 5-fold-diluted Dey-Engley neutralizing broth did not affect the enumeration of the viruses.

### Data analysis.

Viral counts (*N_x_* and *N*_0_) were expressed in log_10_ TCID_50_/25 cm^2^, where *N_x_* is the viral titer recovered from the coated surface and *N*_0_ the titer recovered from the noncoated surface (mean of 3 replicates). The plotted values are mean viral counts ± standard deviations. The limit of quantification (LOQ) of the method was 1.05 log_10_ TCID_50_/25 cm^2^. Nevertheless, in some cases, the LOQ was coating dependent, since cytotoxicity on the cells was observed. The cytotoxicity induced on the cells by the coating solutions was evaluated by swabbing a 25-cm^2^ coated surface (not inoculated with viruses), inoculating on MRC-5 and Vero C-1008 cell lines, and analyzing as described above (virus recovery from noncoated and coated surfaces). Each condition was tested in triplicate. The copper compound-based coating did not induce cytotoxicity, and the LOQ for both viruses was 1.05 log_10_ TCID_50_/25 cm^2^, whereas the QAC-based coating induced cytotoxicity on the two cell lines, increasing the LOQ for both viruses to 3.15 log_10_ TCID_50_/25 cm^2^. Values below the LOQ were entered in the graphs as LOQ with an asterisk. Reduction in infectious virus count (inactivation) was calculated as *N_x_*/*N*_0_ and expressed in log_10_. The statistical significance of log_10_ reductions of HCoV-229E and SARS-CoV-2 obtained with the copper compound-based coating after 50 finger touches (Fig. 4) was determined by using the two-sampled *t* test (unequal variance) using Microsoft Excel for Microsoft 365 MSO. *P* values below 0.05 were considered significantly different.
